# Concise Review: The Potential Use of Intestinal Stem Cells to Treat Patients with Intestinal Failure

**DOI:** 10.5966/sctm.2016-0153

**Published:** 2016-09-16

**Authors:** Sung Noh Hong, James C.Y. Dunn, Matthias Stelzner, Martín G. Martín

**Affiliations:** ^1^ Division of Gastroenterology and Nutrition, Department of Pediatrics, Mattel Children's Hospital and David Geffen School of Medicine, University of California Los Angeles, Los Angeles, California, USA; ^2^ Department of Medicine, Samsung Medical Center, Sungkyunkwan University School of Medicine, Seoul, Republic of Korea; ^3^ Division of Pediatric Surgery, Department of Surgery, David Geffen School of Medicine, University of California Los Angeles, Los Angeles, California, USA; ^4^ Department of Surgery, David Geffen School of Medicine, University of California Los Angeles, Los Angeles, California, USA; ^5^ Department of Surgery, Veterans Administration Greater Los Angeles Health System, Los Angeles, California, USA

**Keywords:** Intestinal failure, Congenital diarrhea, Microvillus inclusion disease, Congenital tufting enteropathy, Intestinal stem cell

## Abstract

Intestinal failure is a rare life‐threatening condition that results in the inability to maintain normal growth and hydration status by enteral nutrition alone. Although parenteral nutrition and whole organ allogeneic transplantation have improved the survival of these patients, current therapies are associated with a high risk for morbidity and mortality. Development of methods to propagate adult human intestinal stem cells (ISCs) and pluripotent stem cells raises the possibility of using stem cell‐based therapy for patients with monogenic and polygenic forms of intestinal failure. Organoids have demonstrated the capacity to proliferate indefinitely and differentiate into the various cellular lineages of the gut. Genome‐editing techniques, including the overexpression of the corrected form of the defective gene, or the use of CRISPR (clustered regularly interspaced short palindromic repeats)/Cas9 to selectively correct the monogenic disease‐causing variant within the stem cell, make autologous ISC transplantation a feasible approach. However, numerous techniques still need to be further optimized, including more robust ex vivo ISC expansion, native ISC ablation, and engraftment protocols. Large‐animal models can to be used to develop such techniques and protocols and to establish the safety of autologous ISC transplantation because outcomes in such models can be extrapolated more readily to humans. Stem Cells Translational Medicine *2017;6:666–676*

**Significance Statement**

The field of intestinal stem cell biology has exploded over the past 5 years with discoveries related to in vivo and in vitro stem cell identity and function. The goal of this review article is to highlight the potential use of these cells to treat various epithelial disorders of the gut and discuss the various roadblocks that will be encountered in the coming years.

## Introduction

Intestinal failure (IF) is a rare multifactorial clinical condition that results in patients' inability to sustain normal growth and nutritional and hydration status without the use of parenteral nutrition (PN) 1, 2. The patients with IF can be classified by whether the small bowel is reduced or of normal length. Short bowel syndrome (SBS) is the most common cause of IF, and it usually results from extensive bowel resection, frequently due to necrotizing enterocolitis, congenital anomaly (e.g., gastroschisis or intestinal atresia), and ischemia from malrotation and volvulus 2. In adults and older children, SBS is commonly associated with Crohn's disease, resulting in severe transmural inflammation that necessitates surgical resection. Therefore, SBS‐associated IF occurs across the age spectrum and is generally associated with prematurity and polygenetic disorders.

In contrast, patients with the non‐SBS form of IF have normal or reduced small bowel surface area despite a normal‐length bowel. A growing number of congenital disorders of the gut epithelium result in a generalized loss of nutrient absorption capacity (Table 1). Among the disorders that alter the villus‐crypt axis and lead to a decline in surface area are microvillus inclusion disease (MVID) 3 and congenital tufting enteropathy (CTE) 4, 5. Several disorders that alter intestinal endocrine cells differentiation or function lead to IF despite a normal surface area 1, 6, 7. Patients with nonepithelial forms of IF also include a broad group of those with severe dysmotility disorders, such as Hirschsprung's disease and chronic intestinal pseudo‐obstruction syndrome, that have various abnormalities in smooth muscle and enteric neuronal cell differentiation or function 7. Similarly, an ever‐expanding number of immunologic disorders, such as IPEX (immune dysfunction, polyendocrinopathy, enteropathy, X‐linked), can lead to IF, and immunomodulatory treatment or bone marrow transplantation are efficacious in a limited number of patients 3. Most non‐SBS forms of IF are caused by a monogenic disorder, which may be amendable to stem cell‐based therapy.

**Table 1:** Causes of intestinal failure due to congenital disorders of the gut epithelium

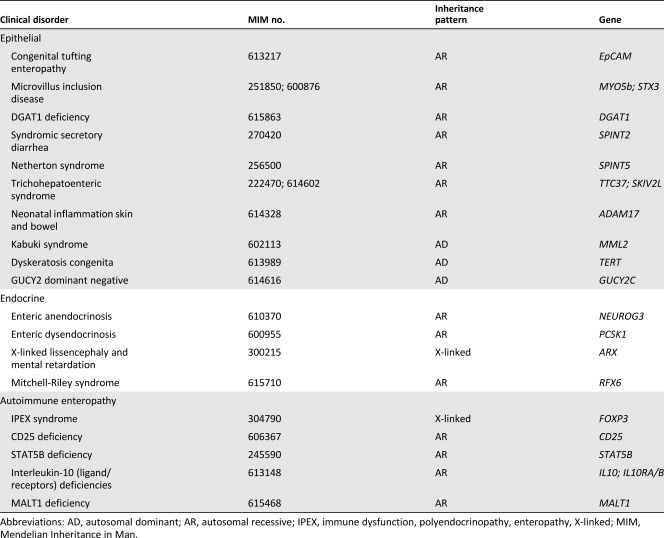

Current management of patients with IF involves close dietary modulation of PN and enteral feeds, medical therapy, and surgical intervention. Parenteral nutrition is the mainstay of therapy for patients with IF; however, its long‐term use is associated with various significant limitations, including IF‐associated liver disease 8, catheter‐related infections 9, and central venous thrombosis 10. In selected patients with specific features that may shorten survival or complicate PN, allogeneic intestinal and/or liver transplantation is potentially a life‐saving therapeutic alternative 11. However, intestinal transplantation is associated with a high mortality; more than half the patients die awaiting transplantation, and the 5‐year survival of patients undergoing transplantation is approximately 60% 12. To mitigate the risk for allograft rejection, lifelong potent immunomodulatory agents with narrow therapeutic index are required; therefore, these therapies not unexpectedly predispose patients to severe recurrent infections and the risk for various post‐transplant malignancies 3, 13. Thus, the development of alternative therapies is needed to improve the long‐term management options in patients with IF.

Intestinal stem cell (ISC) research has made significant strides in improving the understanding of the unique cell populations involved in self‐renewal and differentiation during homeostatic and stressed conditions 14. Various methods can be used to isolate and expand both human somatic ISCs and induced pluripotent stem cells (iPSCs) in intestinal organoids that recapitulate the native epithelium and can be grown on various extracellular matrices in a two‐ (2D) or three‐dimensional (3D) configuration 15, 16.

Concurrent development of gene‐editing techniques has also made treatment of monogenic disorders more likely. Indeed, the first functional restoration experiments have been performed in ISCs derived from patients with cystic fibrosis using CRISPR (clustered regularly interspaced short palindromic repeat)/Cas9 genome editing 17. This corrected disease‐causing mutation indicated the therapeutic potential of combining reconstituted intestinal enteroids and genome editing technology. Gene‐corrected autologous ISC transplantation may be an alternative long‐term solution for patients with IF secondary to defects of the gut epithelium 18, 19, 20. However, several significant hurdles remain before genetically corrected reconstituted intestinal enteroids can be used in patients, including the development of ISC ablation and implantation protocols, and to address safety concerns.

Here we review the current models of stem cell‐driven intestinal epithelial regeneration, with particular emphasis on human models; discuss how advancements in ISCs and gene editing can be applied in management of IF; and address foreseeable obstacles and limitations.

## Intestinal Crypt Stem Cells

**Figure 1:**
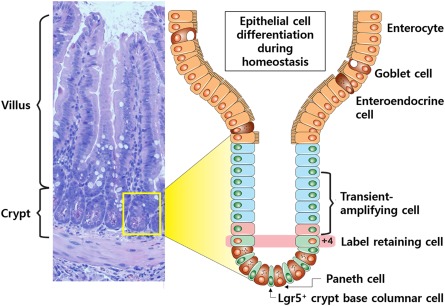
Intestinal crypt. Small bowel epithelium is organized into villus and crypts. Lgr5^+^ crypt base columnar cells are intercalated with Paneth cells at the crypt base and continuously generate rapidly proliferating transit‐amplifying (TA) cells, which occupy the remainder of the crypt. TA cells differentiate into the various functional cells on the villi (enterocytes, goblet, enteroendocrine, and Paneth cells). The +4 label retaining cells (which occupy the fourth position from the crypt base) can restore the Lgr5^+^ stem cell compartment following injury.

The small bowel epithelium is organized into two fundamental structures: villi and crypts (Fig. 1). Villi form functional absorptive units populated by a diverse group of differentiated cells, including enterocytes, goblet, enteroendocrine, tuft, and microfold cells. Each villus is supported by at least six invaginations, termed crypt of Lieberkühn 21. The crypt is occupied mainly by undifferentiated cells, including transit‐amplifying (TA) cells; however, differentiated enteroendocrine and Paneth cells also reside in the crypt. Wedged between Paneth cells are the crypt base columnar cells (CBCCs), which, during homeostasis, maintain both self‐renewal and continuous replacement of the differentiated cells 22.

**Figure 2:**
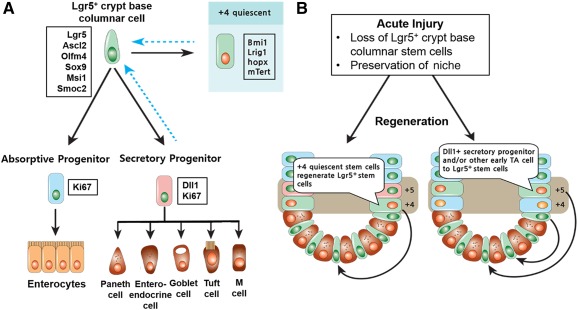
Epithelial regeneration in small bowel. **(A):** During homeostasis, Lgr5^+^ CBCCs at the crypt base self‐renew and differentiate heterogeneous epithelial components. The markers expressed in the cell are indicated in the boxes. **(B):** Acute injury results in the loss of the proliferating Lgr5^+^ stem cells but preserves the relatively resistant Paneth cell precursors, +4 stem cells, and niche cells. Surviving +4 cells function as quiescent stem cells to rapidly regenerate the Lgr5^+^ crypt base columnar cell pool and restore epithelial renewal. Surviving Dll1+ secretory progenitors or other early TA cells fall back into the surviving niche at the crypt base and consequently convert into Lgr5^+^ stem cells to restore epithelial renewal. Abbreviations: M, microfold; TA, transit‐amplifying.

Early observations with ^3^H‐thymidine uptake in the intestinal epithelium suggested two models of multipotent ISCs: CBCCs and +4 label‐retaining cells in mouse models 22, 23. The approach used to reconcile the +4/CBCC debate was to identify a reliable marker that allowed for visualization, isolation, and tracing of ISC progeny. The G‐protein‐coupled receptor, Lgr5, was the first identified specific marker of CBCCs, but additional markers include Ascl2, Olfm4, Msi1, and Smoc2 (Fig. 2A) 24, 25, 26, 27, 28, 29. Genetic lineage tracing in mice harboring GFP–IRES–CreERT2 in the *Lgr5* locus demonstrate that CBCCs are actively cycling and are long‐lived cells that self‐renew and differentiate into the full assortment of epithelial cells 24, 30, 31. Isolated Lgr5^+^ cells were subsequently shown to produce enteroid structures in an in vitro setting, indicating their stem‐like function 32.

Although Lgr5^+^ cells had been established as the "workhorse stem cells" fueling self‐renewal of the intestine, the cells are by no means hard‐wired; they require niche signals and therefore are particularly prone to injury 33. Lineage tracing studies with genes such as *Bmi1*, *Lrig1*, *Hopx*, and *mTert*, suggests that quiescent cells, or a reserve cell population located at the +4 position, are required to reconstitute the CBCCs after injury 34, 35, 36, 37. Specifically, when Lgr5^+^ cells were ablated, crypts remained intact for at least a week through repopulation of CBCCs with quiescent cells 38. Various acute and chronic injuries resulting in the loss of the rapidly dividing CBCCs include radiation exposure, enteric infection, rejection, and inflammation; the surviving progenitor and/or quiescent differentiated populations are thought to revert back to the vacant stem cell niche at the crypt base, where they quickly regain ISC identity and restore epithelial renewal (Fig. 2B) 33, 38, 39, 40. Most research on ISCs and intestinal crypts is from the murine or *Drosophila* models, and a significant gap exists in our understanding of human ISCs.

## Signaling Pathways in Intestinal Epithelial Homeostasis

Ligand‐induced signaling pathways required for intestinal epithelial homeostasis emanate from numerous cell types that constitute the ISC niche. The dynamic interplay between the ISCs and the other cells within the niche creates the system necessary for tissue generation, maintenance, and repair, and for the ultimate design of stem cell therapeutics 41.

### Wnt Signaling

**Figure 3:**
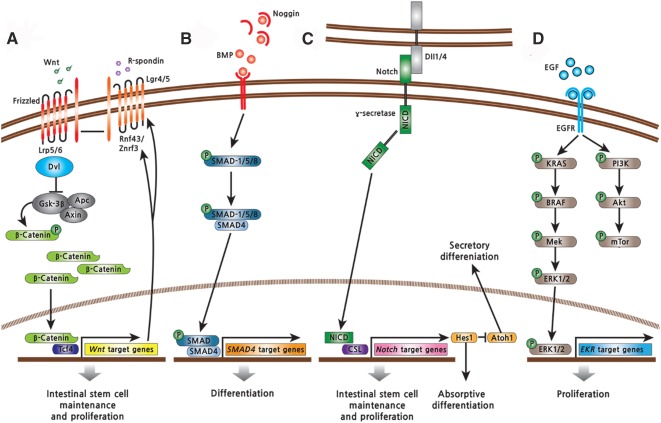
Signaling pathways regulating intestinal stem cells (ISCs). **(A):** Wnt signaling positively regulates the self‐renewal and proliferation of ISCs. **(B):** BMP signaling promotes differentiation of the intestinal epithelium. **(C):** Notch signaling regulates the proliferation of ISCs and differentiation of secretory progenitors. **(D):** EGF signaling regulates the proliferation of ISCs and transit‐amplifying cells by activation of the Ras/Raf/Mek/Erk signaling axis. Abbreviations: BMP, bone morphogenetic protein; CSL, CBF1/Suppressor of Hairless/LAG‐1; EGF, epidermal growth factor; EGFR, epidermal growth factor receptor; NICD, Notch intracellular domain.

In the intestine, canonical Wnt signals from Paneth cells (Wnt3a) and the surrounding mesenchyme (Wnt2b, Wnt4, Wnt5a) act as essential short‐range signals to maintain the ISC compartment 42. Canonical Wnt signaling pathways are activated by the binding of Wnt‐protein ligands to Frizzled‐Lrp5/6 receptors, which pass the biological signal to the protein Dishevelled (Dvl) inside the cell and stabilized β‐catenin. Accumulated β‐catenin enters the nucleus, engaging Tcf transcription factors to activate transcription of Wnt target genes (Fig. 3A). In the absence of a Wnt stimulus, free cytoplasmic β‐catenin displays an exceedingly short half‐life because of the degradation by the ubiquitin/proteasome pathway. Among the Wnt target genes, cyclin D1 and c‐Myc are well‐known drivers of proliferation of undifferentiated cells 21. Wnt signals from Paneth cells and the surrounding mesenchyme fuel the ISC compartment for maintenance and proliferation along the crypt–villus axis 43.

In contrast, several β‐catenin‐independent noncanonical signaling pathways have been proposed wherein Wnt signaling is mediated through Frizzled proteins and receptor tyrosine kinases, such as Ror2, and are independent from the LRP5/6 coreceptor 44. Wnt5a illustrates a Wnt ligand that signals in a noncanonical manner. During the crypt regeneration after tissue injury, Wnt5a augments transforming growth factor‐β (TGF‐β) signaling to restrict epithelial proliferation and promote restoration of the proper tissue architecture through the induction of cell cycle inhibitors, such as p15^INK4B^ 45.

R‐spondins (Rspo) are a family of cysteine‐rich, thrombospondin type I repeat containing proteins that have an essential role in survival and proliferation of Lgr5^+^ cells in in vivo and in vitro settings 46, 47. Mice injected with recombinant Rspo1 and transgenic Rspo gain‐of‐function mice have dramatic crypt cell proliferation and expansion of the villus; this highlights Rspo's potential efficacy for the treatment of SBS 46. Lgr5 and its homologs, Lgr4 and Lgr6, constitute the receptors for Rspo 48. The Lgr5/Rspo complex acts by neutralizing Rnf43 and Znrf3, two transmembrane E3 ligases that degrade the Wnt receptors from the cell surface through ubiquitination 49. Although topical Rspo displayed efficacy in a model of chemotherapy‐induced intestinal mucositis, it is a potent canonical Wnt agonist, and its association with a subset of colon cancers has dampened enthusiasm for its potential therapeutic use in SBS if administered systemically 46, 50.

### NOTCH Signaling

Interaction of a Notch receptor with its cell‐bound ligands, such as Dll1 or Dll4, results in proteolytic release of the Notch intracellular domain (NICD) through the actions of the γ‐secretase protease. NICD translocate to the nucleus, where it binds the transcription factor CBF1/Suppressor of Hairless/LAG‐1 (CSL), thus activating transcription of target genes. Notch signaling activates expression of Hes1, which transcriptionally represses Atoh1, the gatekeeper of entry into the secretory lineage in the gut (Fig. 3B). Simultaneous inactivation of Dll1 and Dll4 factors expressed by Paneth cells resulted in the conversion of proliferating progenitors into goblet cells, concomitant with loss of ISCs 51, 52. In contrast, Wnt signaling represses Notch signaling through direct inhibition of CSL by Dvl 13. ISCs are exquisitely sensitive to Notch inhibition, whereas Notch signaling leads to the differentiation of the absorptive lineage, and an absence of Notch results in secretory lineages 53. Thus, Notch and Wnt signaling pathways are integrated to maintain ISCs and to regulate self‐renewal and cell fate of progeny.

### Bone Morphogenetic Protein Signaling

The bone morphogenetic protein (BMP) signaling pathway acts as a negative regulator of crypts, although the exact mechanism remains unknown. BMP‐2 and ‐4 ligands are expressed in the mesenchyme along the villus 54, whereas BMP inhibitors (Noggin) are expressed in the mesenchyme around crypts 55. The engagement of BMP receptors by BMP leads to complexes between Smad1/5/8 and Smad 4 to repress stemness genes in the nucleus (Fig. 3C) 56. Inhibition of BMP signaling in the villi by overexpression of Noggin results in hyperproliferative and ectopic crypt formation 54.

### Epidermal Growth Factor Signaling

Epidermal growth factor (EGF) signals exert strong mitogenic effects on ISCs and TA cells by activation of the Ras/Raf/Mek/Erk signaling axis (Fig. 3D). Luminal EGF is trophic to the small bowel of parenterally fed rodents, and inhibition of Mek ablates intestinal stem cells 57, 58. Lrig1 is highly expressed by most crypt cells and controls ISC homeostasis by negative regulation of ErbB signaling; its removal leads to expansion of the proliferative compartment 35, 59.

### Summary of Signaling Pathways

In conclusion, both intrinsic and extrinsic signaling controls self‐renewing or asymmetric division of ISCs. Gene‐targeting mice experiments have led to remarkable progress in the understanding of intercellular mechanisms, especially elucidation of the role of the niche in regulating ISC proliferation and directed differentiation. Wnts are provided by Paneth cells and surrounding mesenchyme, whereas Rspo and BMP inhibitors are distinctly provided from nonepithelial sources 60. However, compared with epithelial niche cells, such as Paneth cells, we have an incomplete understanding of the nonepithelial niche cells, including intestinal subepithelial myofibroblasts (ISEMFs), smooth muscle cells of the muscularis mucosa, lymph and vascular endothelial cells, a variety of bone marrow‐derived stromal cells, and enteric nervous system and extracellular matrix (ECM) components, particularly in humans 61.

## Intestinal In Vitro Culture

Long‐term maintenance and propagation of murine intestinal epithelial cells were first reported when the cells were cocultured with native mesenchymal cellular elements in collagen gels in a growth factor‐independent fashion 48. In contrast, the development of a unique serum‐free and mesenchymal‐free in vitro culture system to grow 3D enteroids from a single Lgr5+ CBCC for periods by combining previously defined niche factors (EGF, Noggin, and Rspo1 [ENR]) 32. In addition to culture media supporting the niche signals, laminin‐rich 3D matrix (Matrigel, Corning Inc., Corning, NY, http://www.corning.com) assists dynamic patterning and structural self‐formation of complex organ buds 62.

**Figure 4:**
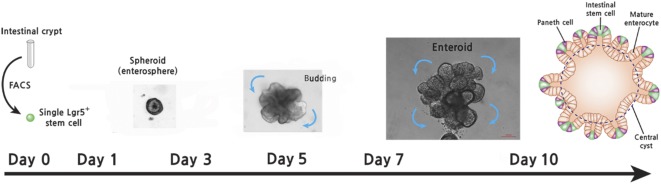
Reconstituted intestinal enteroid culture. Small bowel crypts or Lgr5^+^ stem cells are isolated and plated into a Matrigel plug with culture medium containing epidermal growth factor, Noggin, and R‐spondin. Enterosphere (spheroid) was generated during days 1–4 after initial culturing. After days 5–7, spheroid initiates bud formation and forms reconstituted intestinal enteroid structure (scale bar = 100 μm). Abbreviation: FACS, fluorescence‐activated cell sorting.

The reconstituted small bowel enteroids recapitulate in vivo intestinal epithelial structures: single stem cells initially form thin‐walled, balloon‐like cystic structures (spheroid) and subsequently form multiple‐budding, crypt‐like budding structures (enteroid) 32. Enteroids contain ISCs, progenitors, and the full array of differentiated cells, whereas spheroids consist of more ISCs and progenitor cells and less differentiated intestinal epithelial cells. The location of each cell type in enteroids mimics in vivo intestinal epithelium (Fig. 4).

### 3D In Vitro Culture of Human Intestinal Epithelium

Although murine small bowel epithelium is culturable with the addition of specific niche factors (ENR), additional factors are required for growth of similar human cells. We successfully grew human small bowel epithelium in 3D Matrigel seeded on a feeder layer of ISEMFs with ENR 63. Moreover, an ISEMF monolayer was required to achieve growth of enteroids when implanted subcutaneously in mice 63. The GSK inhibitor CHIR99021 and the E‐cadherin stabilizer Y‐27632 enabled robust enteroid formation from crypts or fluorescence‐activated cell sorting (FACS)‐sorted ISC cell surface markers 64. Others reported that besides the addition of ENR, Wnt3A, nicotinamide, a small molecule inhibitor of Alk, and an inhibitor of p38 were required for long‐term culture of human small and large intestinal epithelium 65. Human reconstituted intestinal enteroids recapitulate digestive, absorptive, and secretory functions 66.

### Fetal Intestinal Epithelium‐Derived Culture

Fetal intestinal epithelium is more acquiescent to in vitro culture than is its adult counterpart. The differentiation of fetal intestinal epithelium depends on developmental age and intestinal region 20. Human and murine fetal intestinal tissue do not form crypts and consist of a series of undulating villi 20. Intestinal epithelium isolated from murine fetal samples forms spheroids and grows independently of EGF or Wnt ligands. In contrast, from postnatal day 15 onward, cultured epithelium forms enteroids that require EGF and Wnt ligands and resemble adult enteroids. Indeed, the enteroids originated from the fetal epithelium retain their unique gene expression pattern after long‐term culture, suggesting that additional exogenous factors are required to mature 20. γ‐Secretase inhibitor and Wnt3A promote murine fetal intestinal spheroids that differentiate into adult‐type enteroids 20, 67. In addition, transplanted murine fetal small bowel spheroids can be engrafted into adult colon and differentiate into colonic epithelium 20, whereas adult small bowel enteroids engrafted into adult colon retain features of the small bowel 19. These findings suggest that fetal intestinal epithelium have a plastic character that differentiate according to niche‐specific signals.

Recently, a novel mesenchymal‐feeder layer‐based culture condition for human fetal intestinal epithelium has been reported to establish homogeneous clonogenic ISC populations, referred to as ground‐state ISCs 68. A combination of ISC niche factors and fibroblast feeder layer preserves the ground state of fetal ISCs. The ground‐state ISCs can efficiently differentiate into villus‐crypt structures when they are transferred to an air‐liquid interface (ALI)‐based differentiation culture condition. Although it remains undetermined whether the ground‐state ISC culture condition can be functional for adult intestinal epithelium 68, 69, the sheet‐like structure of ALI‐differentiated epithelium may provide an advantage for disease modeling and regenerative medicine.

### Generating Intestinal Tissues From Human Pluripotent Stem Cells

Recent advancements with pluripotent stem cells, embryonic (ESCs), or iPSCs have defined the timing of various soluble factors required to generate human intestinal organoids (HIOs) 70. In this system, pluripotent cells are first differentiated into definitive endodermal cells, followed by hindgut endoderm maturation, but the eventual differentiated state of the epithelium is more characteristic of early fetal tissue 71, 72, 73. Some have speculated that the reasons for the immaturity and diminutive size may be the insufficient time that the cells have to mature and/or limitations of oxygen and nutrient diffusion 70, 71. To improve the maturation of epithelium derived from pluripotent‐derived HIOs, these cells were engrafted under the murine kidney capsule to enhance vascularization by host endothelial cells, and after 6 weeks, HIOs grew significantly larger than controls 74. Moreover, the HIOs had more of a mature adult‐type crypt‐villus architecture, differentiated intestinal cell lineages, and subepithelial layers. Furthermore, ileocecal resection induced the expansion of crypts and villi in engrafted HIO‐derived tissues 75. Functional xenograft transplantation could be a possible approach that promotes the necessary maturation and differentiation of pluripotent HIOs to model pathologic conditions that manifest in a nonfetal phenotype.

## Intestinal Stem Cell Therapy for Intestinal Failure

Stem cell therapy is a promising alternative strategy for overcoming the current limitations of IF treatment, but genetic modification of ISCs will be necessary to fully exploit their potential. Through use of recent culture technology, gene editing of ISCs is enabled by the highly amenable and reproducible culture of intestinal epithelium. The clonogenic capacity and rapid expansion of spheroids should permit culture of genetically identical populations of intestinal epithelium. However, optimal methods to expand human ISCs, ablate native in situ ISCs while maintaining a receptive niche, and optimally engraft genetically manipulated ISCs have yet to be described. If successful engraftment of modified ISCs into the ablated small bowel niche of IF patients is possible, autologous ISC transplantation will be a promising treatment option.

### Gene Editing in Intestinal Enteroids

Although numerous viral and nonviral gene‐editing systems have been developed, optimization has not been described for the use of human ISCs in gene therapy 76. Genome‐integrating viral vectors are usually characterized by highly efficient and long‐term transgene expression, and while safety concerns do exist, current lentiviral constructs have a low frequency of integration into sites that predispose to cancer 77. Spheroids can be transduced by lentiviruses, thereby allowing manipulation of genes within these cells, but the transduction of enteroids is very limited 78. Although nonintegrating viruses, such as adenovirus, are also highly efficient in transduction and are safer, they offer only a limited duration of transgene expression 79.

Recently, genome engineering with site‐specific nucleases to edit DNA allowed targeted gene knockouts, generated tissue‐specific cell lineage reporters, overexpressed genes from defined loci, and introduced and repaired point mutations in stem cells 80. The CRISPR/Cas9 site‐specific nuclease approach for genome editing has dramatically advanced in the past year and will likely be the favored approach for gene editing in the coming years 81. Indeed, CRISPR/Cas9 editing was used to correct the *CFTR* locus by homologous recombination in cultured ISCs of cystic fibrosis patients 17. The corrected allele is expressed and fully functional, as measured in clonally expanded enteroids. This study provides proof‐of‐concept for gene correction by homologous recombination in ISCs derived from patients with a monogenic defect. However, two main challenges remain for the use of genetically corrected reconstituted intestinal enteroids in clinical application: the development of ISC ablation and engraftment techniques and the resolution of the safety concerns.

### Intestinal Enteroid Transplantation in the Small Bowel

The prerequisite for ISC therapy for intestinal disorders is successful engraftment of implanted cells. The proof‐of‐concept that donor ISCs can reconstitute a new intestinal epithelial layer after ablation was first demonstrated in 2005 when transplantation of ileal organoids containing crypts and surrounding mesenchyme was successfully engrafted into a denuded jejunal segment 82, 83. In adult rats, a long segment of jejunum was isolated, with its blood circulation left intact, and the enterocytes were partially ablated by using luminal high‐velocity perfusions with EDTA solutions. Ileal organoids were harvested from neonatal rats and successfully transplanted into the ablated segments to generate a neoileum. The recipients subsequently underwent resection of the native ileum, and the neoileum was anastomosed in its place. The neoileum exhibited enhanced taurocholate uptake and ileal bile acid transporter expression when compared with the native jejunum. Indeed, the ileal organoid transplantation was capable of treating a model of bile acid malabsorption 83, 84. To date, this is the only description of organoid engraftment into the small bowel, highlighting the technical challenges of engrafting ISCs in the mouse.

### Reconstituted Intestinal Enteroids Transplantation in the Colon

More recently, single murine Lgr5^+^ ISC‐derived colonoids were transplanted into dextran sulfate sodium (DSS)‐ablated colonic epithelium of Rag2^−/−^ immunodeficient mice 18. The implanted colonoids constituted a single‐layered epithelium, which formed self‐renewing crypts that were functionally and histologically normal with long‐term engraftment. The colonoid transplantation accelerated the recovery of epithelial barrier function and reversal of inflammation in the DSS colitis model. Moreover, murine fetal small bowel‐derived spheroids were successfully engrafted into immunodeficient mice with DSS‐induced colonic damage 20. Indeed, transplanted murine fetal small bowel‐derived spheroids were incorporated into the regenerating colonic mucosa and differentiated into colonic epithelium, underscoring the notion that fetal ISCs have a plasticity that enables cross‐lineage differentiation into cells of engrafted intestine. Moreover, adult mouse small bowel‐derived enteroids were successfully transplanted into mechanically disrupted rectal mucosa of immunocompetent mice and retained small bowel features that were distinct from the colonic epithelium 19. This interesting finding suggests that the residual colon could be used to engraft small bowel ISCs in patients with SBS, thereby enhancing nutrient absorptive capacity. These studies provide preliminary evidence that reconstituted intestinal enteroids could be used as a source for ISC therapy of intestinal disorders, maintaining their identity along the gut through an epithelium‐intrinsic mechanism 19.

## Prospects of ISC Treatment for Monogenic Enteropathies Resulting in IF

The replacement of diseased epithelium with gene‐corrected autologous ISCs or human leukocyte antigen‐matched allogenic ISCs would be feasible therapeutic approaches for IF with severe monogenic enteropathies. Overexpressing the wild‐type form of the defective gene may be sufficient for treatment. MVID and CTE are among several monogenetic disorders that would be feasible to pursue.

MVID is an autosomal recessive disorder characterized by chronic profuse watery secretory and malabsorptive diarrhea 3. The small bowel mucosa demonstrates villus atrophy with crypt expansion without inflammatory infiltrate. The most common form of MVID is associated with biallelic mutations of the *MYO5B* gene, which codes for a nonconventional motor protein; the loss‐of‐function variants result in abnormal intracellular protein trafficking and defects in recycling of apical vesicles and have abnormalities of cell polarity. Recent whole‐exome sequencing from patients with a milder form of MVID showed biallelic loss‐of‐function mutations in *STX3*, which is the encoded cellular receptor for transport vesicles in enterocytes. In vitro assessment of patient‐derived enteroids and overexpression cells of truncated STX3 recapitulated most characteristics of variant MVID 85.

Similarly, patients with CTE present with persistent neonatal diarrhea that is both secretory and malabsorptive in nature. Biopsy of the small bowel demonstrates focal epithelial “tufts,” with villus atrophy and crypt expansion. CTE is associated with biallelic loss‐of‐function mutations of the *EpCAM* gene, which encodes an epithelial cell adhesion molecule protein, resulting in isolated congenital diarrhea without associated extra digestive symptoms. Specific founder mutations associated with consanguineous pairing of families from the Arabic peninsula and Mexico are particularly common in patients with CTE and is in contrast to MVID, which is associated with significant allelic variant heterogeneity 4, 5.

**Figure 5:**
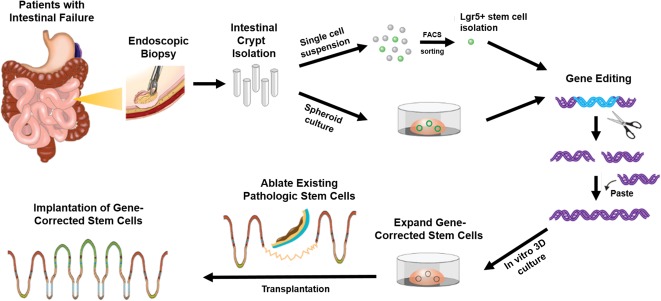
Outline of intestinal stem cell (ISC) transplantation as a treatment for patients with intestinal failure caused by monogenic disease. The intestinal crypts can be isolated from the mucosa of patients by using endoscopic biopsy. Isolated crypts are dissociated into single cell suspension, and Lgr5^+^ ISCs are identified by using FACS. In an alternative method, ISCs can be obtained by spheroid, which consists of ISCs and its progenitors. Gene editing is intended to restore the function of ISCs, including the overexpression of the corrected form of the defective gene or selective correction of the disease‐causing mutation. The gene‐corrected ISCs are expanded in an in vitro 3D culture system and transplanted into the segment of the patient's small bowel, where pathologic ISCs are ablated. Successfully engrafted ISCs can differentiate and reinstate all lineages of epithelium, with normal absorptive and secretory function. Abbreviations: 3D, three‐dimensional; FACS, fluorescence‐activated cell sorting.

An obvious approach for ISC gene correction of monogenic disorders, such as MVID or CTE, would be ex vivo isolation, correction, and expansion of ISCs and subsequent implantation (Fig. 5). Specifically, intestinal crypts could be isolated from the mucosal biopsy specimen of patients using endoscopic biopsy or short segment of intestine using minimal invasive surgery. ISCs may be isolated from crypts by FACS or can simply be expanded in vitro by using elevated canonical Wnt signaling that promotes the relative abundance of ISCs. The genetic defect would be edited by using a CRISPR/Cas9‐type approach, and the gene‐corrected ISCs would be expanded in vitro and subsequently engrafted into the segment of the patient's small bowel where the native ISCs have been ablated. Successfully implanted ISCs should be capable of differentiating into all lineages of epithelium, and normal absorptive and secretory function would be recovered.

Another promising approach may the use of gene transfer mediated by a recombinant adenovirus‐associated viral (AAV) vector 86. AAVs are nonenveloped, single‐stranded DNA viruses that replicate only in the presence of helper virus; they are not currently known to cause disease 87. The efficiencies of AAV‐mediated gene targeting varies significantly on the basis of the species, cell type, and AAV capsid serotype. This AAV‐mediated gene targeting has enabled diverse genomic modifications both in vitro and in vivo, raising the possibility of gene editing of ISCs while they remain in situ, thereby obviating the ex vivo phase of isolation, correction, and engraftment 87. These features make AAVs an attractive candidate for correcting monogenic gastrointestinal disorders, particularly if the corrected ISCs have a growth and survival advantage when compared with the native ISCs.

The use of AAV for gene therapy is illustrated by its efficacy in many clinical trials, including those for hemophilia B 88, Leber's congenital amaurosis type II 89, choroideremia 90, and lipoprotein lipase deficiency 91, with the latter obtaining regulatory approval in the European Union in 2012. Indeed, to date, AAV vectors have been used in more than 162 clinical trials worldwide 92. While clinical trials using AAV for gene therapy of MVID and CTE may be an attractive option, AAV vector‐mediated gene transfer must be assessed for the most efficient AAV capsid serotype that transduces human ISCs.

## Current Challenges for Intestinal Stem Cell Transplantation

Although rodent models have demonstrated the feasibility of intestinal stem cell‐based therapies, several gaps and hurdles need to be addressed to optimize the clinical utility of these therapies, particularly for the treatment of monogenetic disorders. For instance, we need to improve our understanding of human ISC and progenitor cell biology, while establishing optimal ablation and engraftment techniques in a large‐animal model, as we continue to collect preclinical evidence of safety and therapeutic efficacy.

First, many of the lessons learned from rodent studies do not necessarily translate to human systems. Because most studies of ISCs and their niche were conducted in mouse models, we have a relative incomplete understanding of the human ISCs/progenitor and niche cells. Certainly compared with the mouse, in vitro culture for human cells requires additional growth factors and small molecules, suggesting that the biological properties of human cells are more complex. Among the shortcomings are the absence of selective markers that identify the various nondifferentiated cell populations, both for fluorescence‐activated cell sorting and immunofluorescence 64. We still need to develop lineage tracing techniques for our human models. In addition, although we have an inadequate understanding of the nonepithelial niche cells in mice, certainly the human intestinal niche has barely been explored. Substantially more research is needed to understand the intricacies of these human cells before stem cell‐based therapies can be appropriately optimized.

Second, to date, regenerative approaches for diffuse intractable diseases have focused on the development of stem cell transplantation. Certainly a prerequisite is a detailed procurement plan to obtain a sufficient number of stem cells because a robust expansion that is sufficient to repopulate the immense surface area of the small bowel has not been described for adult human ISCs. Furthermore, although ISCs can be expanded ex vivo in the presence of growth factors, recombinant proteins are exceedingly expensive. Certainly it may be worth adapting recently methods described for in vitro expansion of human pancreatic β cells generated from iPSCs that use a renewable cell source and a scalable method 93.

Third, efficient methods to enrich for subtypes of ISCs are lacking, including those isolated from different regions of the gut or from different developmental ages that might exhibit varying responses to signaling cues that could affect their expandability.

Fourth, robust methods to integrate engrafted ISCs into existing intestinal mucosa are still underdeveloped. Previous reported experimental ISC transplantation models in rodents have applied different techniques, and for use in humans these methods need to be both safe and carefully standardized. The first step of transplantation is preparing the diseased ISC for ablation and generating a receptive niche to engraft healthy ISCs. EDTA sequesters divalent cations, resulting in the separation of epithelium from its underlying basement membrane, and can be used with soft serosal compressions or luminal brushings to enhance the extent and uniformity of epithelial separation along the gut 84. However, luminal EDTA may be absorbed and can induce significant immediate side effects, including hypotension, electrolyte imbalance, and paresthesia. If the intestinal surface area required to maintain enteral nutrient balance can be estimated, surgical or endoscopic mucosal excision, endoscopic laser therapy, or photodynamic therapy might be alternative options 94. Although these mucosal ablation approaches are invasive procedures, other novel techniques, such as minimal radiation or the use of detergents and/or cytotoxic agents, could be explored. Therefore, large‐animal‐based preclinical studies will certainly be required, because animals such as pigs and nonhuman primates resemble humans with regard to size, an important limitation of current rodent models 95.

Fifth, ensuring the safety and efficacy of stem cell‐based products is a major challenge. Cells manufactured in large quantities outside their natural environment can potentially become ineffective and induce adverse effects, such as tumors or immune responses 96. Moreover, autologous stem cells with gene editing may enhance such risks. The U.S. Food and Drug Administration (FDA) and the national regulatory agencies in other countries may have more stringent precautions, far more so than for many other regulated products 96.

Finally, the FDA will not allow the use of cells that were maintained in Matrigel because it is a poorly defined extract from an Engelbreth‐Holm‐Swarm tumor in mice. Of course, Matrigel mimics the native basal membrane composed of ECM molecules, such as laminin, collagen type IV, entactin, and heparan sulfate proteoglycans, as well as some growth factors, such as TGF‐β and fibroblast growth factor 97. We have used collagen and various laminins in 2D and 3D culture for reconstituted intestinal enteroids, but the culture efficiency is lower, and several of these ECMs are expensive 18. Certainly hydrogels might be a possible solution to this problem.

Therefore, among the current limitations of using ISCs for regenerative medicine is the need to establish a large‐animal model in which the techniques and safety of transplantation methods may be established, including methods of mucosal ablation, and the dosing of implanted cell, optimal ex vivo expansion of ISCs, and the routes of administration may be extrapolated readily to humans.

## Conclusion

IF is a rare but life‐threatening condition resulting from the inability to maintain the growth and development with nutrition obtained via the enteral route. Although PN and allogeneic intestinal transplantation improve the survival of patients with IF, current therapies have significant long‐term limitations. The recent development of methods for expansion of human ISCs and iPSCs has made it possible to reconstitute complex cellular structures that mimic the native bowel in an in vitro setting. Moreover, when intestinal enteroids were transplanted into suitable donor sites, they gave rise to a complete mucosal epithelium with all cell lineages, and the appropriate ECM enables the growth potential of the neo‐mucosa. Finally, the development of genome editing techniques, such as CRISPR/Cas9, has made genetic engineering of stem cells possible and provides new opportunities to perform autologous stem cell transplantation for IF with severe monogenic enteropathies. Although the methods used to ablate diseased mucosa, dosing and optimal growth of ISCs, and the route of administration still require optimization, the replacement of diseased epithelium with corrected epithelium originating from gene editing of autologous stem cells is a feasible therapeutic approach.

## Author Contributions

S.N.H., J.C.Y.D., M.S., and M.G.M.: manuscript writing, final approval of the manuscript.

## Disclosure of Potential Conflicts of Interest

The authors indicated no potential conflicts of interest.
